# Evidence-based treatment for schizophrenia: a personal perspective

**DOI:** 10.1017/S1092852924000555

**Published:** 2024-10-28

**Authors:** Bethany Yeiser

**Affiliations:** President of the CURESZ Foundation, Fairfield, OH, USA

## Abstract

My name is Bethany Yeiser, and I am an individual living with schizophrenia. My schizophrenia has been in full remission since 2008, thanks to treatment with clozapine, the vastly underutilized medication for refractory schizophrenia.

My name is Bethany Yeiser, and I am an individual living with schizophrenia. My schizophrenia has been in full remission since 2008, thanks to treatment with clozapine, the vastly underutilized medication for refractory schizophrenia.

My journey through schizophrenia and finding my way back was not easy.

I was born in 1981 to a loving family. My childhood was wholesome and happy. At age 7, I began studying violin, which quickly became my passion. I began practicing for 4 hours a day at age 13 and was accepted as a student of a violin professor at the Cleveland Institute of Music that same year.

I also excelled academically. At age 15, I discovered Ohio’s Post-Secondary Educational Options Program, allowing high school students to take classes at local colleges and universities for dual high school and college credit. After attending Lakeland Community College for 2 years, I graduated from high school with 2 years of college credits and a 4.0 GPA.

My dream school was the University of Southern California, as I was attracted to their music program. However, after visiting USC, I realized how many other exciting academic options were available to me and soon settled on a bachelor’s degree in biochemistry and molecular biology.

In 1999, at age 17, I traveled from Ohio to USC. I would live in the honor’s dormitory, having been awarded a half-tuition scholarship. From the beginning, I was extremely busy with difficult classes including organic chemistry. I was happy to land a position in a research laboratory during my first semester, studying enzymes that replicate DNA (which had important implications in certain human cancers). I also auditioned to become concertmaster of USC’s community orchestra and won.

But something was wrong. I remember wanting nothing to do with the other students, usually eating alone and never going to social functions or outings. I committed to attending a local church but rarely mingled. I would arrive late and leave the minute it was over to rush back to the lab.

During my junior year of college, I began to develop a deep-seated urge to change the world, leaving a great legacy and impact. At this time, my church was sponsoring a small group of young women to visit a remote and impoverished community in China.

I left for China during the winter break of my junior year of college. While there I remember thinking: Can I change the lives of a million people in China? Or Millions? Something inside of me said yes, this was possible, and that it would happen immediately.

Looking back, I understand this was delusional thinking. If I wanted to impact a large number of impoverished Chinese people, there was the need to pursue higher education, perhaps a PhD in economics or political science. I would have needed to study Chinese for years and live in China for some time to establish relationships and credibility. Instead, I believed my next step forward was to visit Africa, to live among a different culture of people in need. During the summer of 2002, prior to the start of my senior year at USC, I spent 2 months living in Nairobi. The church in Cleveland where I had grown up sponsored me and paid nearly all my expenses.

About this time, I developed a symptom of schizophrenia I had never heard of, dromomania. This is an uncontrollable urge to travel.

Upon my return from Africa, I began planning a trip to Thailand to visit an American family I knew there. But suddenly, I was unable to focus, even struggling to pass my classes. My parents were at a loss to understand what was happening in my life. But they knew who I was, and how much my degree meant to me. They were entirely certain that if they told me they might withdraw their funding if I continued with my travel plans, I would cancel my trip. However, my psychiatric physician looks back on this time as the onset of my first psychotic break. Convinced that traveling to Thailand was more important to my future than my college degree, I told my parents I was going to Thailand. Then, I refused all contact with them for the next four and a half years, during which I descended into a life of psychosis and homelessness. I believed that my parents would try to prevent me from making a worldwide impact. I thought my travel around the world was commanded by God and saw my parents as adversaries.

I soon dropped out of USC officially and lost my dorm room, refusing all help from friends and family, paranoid they would stop me from making my worldwide impact. I had maxed out my credit cards in Thailand and rapidly ran out of money.

In March 2003, I became homeless. Nonetheless, I quickly became an expert at washing up in public bathrooms and carrying only light changes of clothing to appear like a student with a backpack. I would stay most nights and sleep in the USC library. I actually made some friends in the library with engineering students who regularly wrote code late into the night.

I prevaricated about my status as a student, telling acquaintances that I was a part-time graduate student. Had I been in my right mind, I very likely would have been a part-time graduate student with an interest in international affairs on the side. I told myself my lies were acceptable since, once I became a successful billionaire, no one would care.

In my free time, I started studying ancient Hebrew at a library every day and, after a few months, I developed a basic proficiency. I also made friends with a computer engineer and his wife from mainland China who taught me how to chat in Mandarin Chinese, until my paranoia surfaced, and I became too afraid of them to continue free lessons. To this day, in 2024, I retain my ancient Hebrew proficiency and have basic conversational skills in Chinese.

But about 3 years later, on January 28, 2006, again, everything changed. I was sitting on a park bench on the university campus in the early afternoon when I heard voices in my head for the first time, insulting me, yelling at me, and then changing to a positive tone and complimenting me. I immediately realized something was not right. However, I thought through mental illness and decided that I was too strong, too smart, and too normal to ever be mentally ill. I concluded that what I was experiencing must be normal and, since no one else was talking about it, I should not either.

About that time, I gave up hiding in libraries and lounges on the USC campus, afraid I would be caught. My backup plan was a local churchyard. I would stay there for the next 13 months.

With time, my hallucinations became more problematic. I heard voices in my mind which I knew no one else could hear, as well as voices and noise (such as cars passing by or birds chirping) in my reality. When hearing noise in my reality, I was entirely unaware if these were real sounds or hallucinations. One day I looked in a mirror, but my face resembled the face of the character Lisa from the show The Simpsons.

My hygiene plummeted as I stopped washing up in public restrooms. It was hard to do it quickly, and I always was afraid someone would walk in on me while I had one of my feet in the sink. At this time, I also became shameless about rummaging for food to eat in garbage cans on the streets of LA, even when other people noticed, because I was so hungry.

On October 14, 2006, my twenty-fifth birthday, the voices pressured me relentlessly to go back to the campus and spend the night in a lounge where I had stayed several times and never been caught. I went there again 2 days later, dirty, and clearly not belonging on the campus. I remember looking out the window and seeing Los Angeles Police Department officers, which I had never seen on campus before. They were there to pick me up, and I was taken to jail for about 3 days.

In the back seat of the police car, I asked the officers if they would take me home afterward (which to me was the campus area). They said yes, of course. I did not know they were being sarcastic.

When I entered the jail, they asked me if I needed medical attention. I saw a large room with women getting shots. Since they needed medical attention, I wondered why they were in jail.

My jail cell was a tight fit for 2, but there were 4 of us in it. When they took my fingerprints, I was terrified the ink would give me a terrible disease. Soon, I was transferred to a big room that was literally as dark as a cave. During my life I had never been afraid of the dark before but in jail, I was. We had a 15-minute break later with some translucent light coming in from the roof and then were returned to the darkness. I recall one good thing about the jail—I greatly appreciated the hot food they served us.

The morning of the third day, I was taken to a courthouse where I signed paperwork declaring I would return for my day in court, though I had no way of getting there and was embarrassed to show up dirty and with shoes that had almost completely fallen apart.

You would think that my traumatic experience in jail would have changed my life—that I would have finally called family or friends and asked for help. But I did not. I believed God wanted me to wait in the churchyard. I believed a billionaire was coming and could enter my life at any time. I resolved to wait for him. The judicial and social service systems had failed me. I resumed sleeping outside, homeless, and in need of medical assessment and treatment.

In November 2006, the day before Thanksgiving, I was unable to read a newspaper and decipher the date but guessed it was a week before Thanksgiving. I always left the churchyard early in the morning and never returned until evening, but that day, because my hallucinations were so intense and bothersome, I went to the churchyard in the afternoon.

A couple of police officers called out to me asking where I lived. When they did not believe the lie everyone else seemed to believe, they picked me up and took me to jail a second time. Many people stayed at that churchyard. To this day, I do not know what my crime was that afternoon.

My second experience in jail was much different than the first. We had clean uniforms, and there was more natural light. At times, I was in intensely crowded spaces for hours, as we were shuttled to different parts of the building. But after 5 days, one morning, I woke up thankful for the jail. I was released a few hours later. Looking back, again, I see the failure of the systems of judicial and social services. My life outside, homeless, had become so difficult, jail did not seem so bad.

March 3, 2007, was my fourth-year anniversary of becoming homeless, though I did not know the date. The voices were severe, the most irritating stimulus I could ever imagine, forcing me to scream profanity, though I had never used profanity in my life prior to hearing the voices. Suddenly, a police officer I had not seen snuck up to me and pulled my hands behind my back. When I was told I was being taken to a psychiatric ward, I was actually happy to hear it. I expected to be released immediately.

Being involuntarily hospitalized was a terrible experience. I resented my doctor and was absolutely certain I was not sick. But on my first medication, which I was mandated to take, the visual hallucinations, delusions, and paranoia disappeared. When my parents visited, still interested in a relationship, I wondered why we were not already in touch. They had never done anything wrong, and after the medication had cleared my mind, I understood that.

I left the hospital with no paranoia, no delusions, and no visual hallucinations, but the voices I heard inside my mind were still there, which led me to believe the medication was not helping at all.

What I needed was an education about my illness and medication. I needed someone to point out to me the changes in my behavior and clarify that these changes were directly due to the antipsychotic medication. However, instead, I was discharged from the hospital without knowing my diagnosis, what it meant, or what the pills were for. I also was unaware that my psychiatrist had told my parents that I was permanently and totally disabled, which meant that I would never work or attend college again, or live independently.

I flew to Cincinnati with my parents, as they had moved there from Cleveland while I was in California. They gave me a beautiful room, invited me to meet their friends, and encouraged me to walk through their vibrant community.

My antipsychotic side effects soon became unbearable. I had akathisia, an extreme restlessness that never went away, as well as a flat effect. I found myself sleeping 16-18 hours a night, and developed a ravenous appetite, quickly gaining over 15 pounds. Because I did not believe I needed this medication, and thought it was ruining my life, I did the logical thing, and discontinued my pills. At first, as the side effects went away, I felt great. However, within about 2 weeks, my command hallucinations were back, causing me to scream and shout profanity. I was soon hospitalized for a second time.

During my first Ohio hospitalization, I still did not receive information about my diagnosis and symptoms, or how my medication worked. However, a physician sat down with me and told me what I believe was the most important thing I needed to know: if you go off your antipsychotic and on it again, it can be less effective, even at higher dosages. He explained to me that this is what leads to disability. That day, I was convinced to always take my medication, and I have taken my antipsychotic now without interruption for 17 years.

The next 12 months were the hardest of my life, as I was given 5 different atypical antipsychotics, and sometimes combinations of 2 of these antipsychotics. I thought my first 3 doctors were correct, that I would be permanently and totally disabled. But just when everything seemed hopeless, I was referred to a new psychiatrist, Professor Henry Nasrallah, MD, a renowned schizophrenia expert who started me on clozapine, the only antipsychotic approved by the FDA for treatment-resistant schizophrenia. Within 4 to 6 months, the auditory hallucinations disappeared, and I fully recovered.

Following my recovery, in 2009, I enrolled at the University of Cincinnati and graduated with honors in molecular biology (3.84 GPA). Over the following 2 years, I wrote and published a memoir called *Mind Estranged: My Journey from Schizophrenia and Homelessness to Recovery*, about the journey of my recovery. Today I work both as President of the CURESZ Foundation and as a national motivational speaker, frequently traveling around the United States. I share my story of full recovery, though I was repeatedly told my recovery would be impossible. Some people have told me I am the exception to the rule. This is what led me recently to publish another book, together with Dr. Henry Nasrallah, that we called *Awakenings: Stories of Recovery and Emergence from Schizophrenia*, featuring 28 recovery stories of individuals who have made remarkable recoveries after being diagnosed with schizophrenia.

There are many changes we need to see in the mental health-care system of the United States. The most urgent in my opinion is the creation of a new standard of commitment for involuntary hospitalization, and it needs to be easier to require psychiatric evaluations. Looking back, I should never have been allowed to struggle on the streets outside for 13 months before I was taken to a hospital for evaluation. Months before I was committed, had I seen a doctor, he probably would have recommended an involuntary hospitalization. But I was neglected, and no one took me to see a physician for evaluation and treatment. And because of the high standard for involuntary commitment, my parents were powerless.

In our country, in order to be involuntarily hospitalized, a person must be a danger to self or others or gravely disabled (which I finally became in 2007). Many families have loved ones who are desperately sick but do not meet these difficult criteria. Some parents will take their acutely psychotic son or daughter to a hospital, but because they are not “sick enough,” they are turned away. The parents have to simply wait for their loved one to get worse in order to qualify for admission to a psychiatric hospital, unless they get arrested and end up in jail as a criminal.

It is absolutely essential to give people as much autonomy as possible, but those who are paranoid, delusional, and experiencing hallucinations often are totally unaware they are sick and will refuse help that could treat their psychotic symptoms and greatly improve their lives. Many of these people with serious psychiatric brain disorders descend into homelessness, as I did, and their quality of life becomes very poor. Unfortunately, many more of these people, like me, will end up in jails or prisons, though psychosis influences their behavior and their real need is for a hospital where they can receive medical treatment. In the United States, it is much easier to become homeless or be jailed than it is to be involuntarily hospitalized for psychiatric and medical assessment and treatment.

Perhaps I was one of the lucky ones, only incarcerated twice for about 8 days in total. Looking back, I realize how easy it is for a psychotic individual to commit a pretty crime. I also realize that I would never have consented to a life-changing involuntary hospitalization, but because of it, I was able to redeem my life. It placed me on the path to the functional success I enjoy today.

Education is vitally important. People struggling with conditions such as breast cancer or diabetes are given pamphlets, stuffed animals, other gifts, information for support groups, and told what to do if they have medication side effects. People with schizophrenia, on the other hand, are often left with very little information, or none at all, and a bottle of pills they can choose to take or discard. It is rare to offer first-episode patients, or any patients, long-acting injectables. This is unfortunate because those formulations are highly effective at preventing relapse due to poor adherence to pills.

In our society, we do not see children or elderly persons with dementia living on the streets. If a child is facing homelessness, social services come to their rescue and bring them to a place where they have housing, clothing, food, and education. A person struggling with Parkinson’s disease cannot simply refuse medication and choose to live underneath a bridge. Our legal system is set up such that many of the neediest among us, who cannot make rational choices on their own, or choose to get help, are not simply left to suffer. But when it comes to schizophrenia, severely psychotic persons are largely unprotected and forgotten, or end up jailed, homeless, or prematurely dead.

Recently, in Cincinnati where I live, a social worker saw a woman at a church event whom he had previously met when she was homeless. She looked groomed and well, and he approached her to ask what had happened. She looked at him and said, “Why? Why did you leave me outside for such a long time?” Following her involuntary hospitalization, she was able to begin a new life, and today, she does not understand why no one brought her to a hospital much sooner.

I wish I had never dropped out of USC and that my homelessness had never happened. I also wish that I had never been driven by insanity to hide on the campus in libraries and lounges for years, waiting for an imaginary person. I wish I had never been taken to jail for trespassing on a campus where I was once so excited about attending classes and scoring A’s. But today, through sharing my story, I hope to be instrumental in helping more needy Americans get the treatment they need. We need to get them off the streets and keep those who need treatment out of our jails and prisons. Most importantly, we need to offer the most vulnerable among us the hope and potential recovery that comes with effective treatment for psychiatric brain disorders.

Due to my experiences, as well as my desire to prevent others from having to go through something similar, I serve as President of the CURESZ Foundation, which I founded in 2016 with the psychiatrist who brought me to full recovery, Henry Nasrallah, MD. CURESZ stands for Comprehensive Understanding via Research and Education into SchiZophrenia. The CURESZ Foundation provides education, advocacy, and information about cutting-edge and underutilized treatments for schizophrenia such as clozapine for treatment resistance, long-acting injectable medications for relapse prevention due to nonadherence, and new medications for movement disorder tardive dyskinesia. We offer support for families including a caregivers’ mentoring program, student-based clubs, a support group, and a wide range of educational videos.

